# The Influence of Chronic Ego Depletion on Goal Adherence: An Experience Sampling Study

**DOI:** 10.1371/journal.pone.0142220

**Published:** 2015-11-12

**Authors:** Ligang Wang, Ting Tao, Chunlei Fan, Wenbin Gao, Chuguang Wei

**Affiliations:** 1 Key Laboratory of Mental Health, Institute of Psychology, Chinese Academy of Sciences, Beijing, China; 2 The Core Facilies of Institute of Psychology, Chinese Academy of Sciences, Beijing, China; University of Leeds, UNITED KINGDOM

## Abstract

Although ego depletion effects have been widely observed in experiments in which participants perform consecutive self-control tasks, the process of ego depletion remains poorly understood. Using the strength model of self-control, we hypothesized that chronic ego depletion adversely affects goal adherence and that mental effort and motivation are involved in the process of ego depletion. In this study, 203 students reported their daily performance, mental effort, and motivation with respect to goal directed behavior across a 3-week time period. People with high levels of chronic ego depletion were less successful in goal adherence than those with less chronic ego depletion. Although daily effort devoted to goal adherence increased with chronic ego depletion, motivation to adhere to goals was not affected. Participants with high levels of chronic ego depletion showed a stronger positive association between mental effort and performance, but chronic ego depletion did not play a regulatory role in the effect of motivation on performance. Chronic ego depletion increased the likelihood of behavior regulation failure, suggesting that it is difficult for people in an ego-depletion state to adhere to goals. We integrate our results with the findings of previous studies and discuss possible theoretical implications.

## Introduction

The capacity for self-regulation is essential to many aspects of life, from individual concerns such as diet and bodily exercise to social concerns such as inhibiting addictive behavior and avoiding violence. However, failure of self-regulation is frequent, notably among people struggling with obesity, drug abuse, violent crime, eating disorders, and certain chronic diseases (e.g., cancer and heart disease) [1–3]. Research suggests that weakened control mechanisms caused by recent assertions of self-control increase the likelihood of self-control failure [4].

Baumeister and his colleagues developed a strength model of self-control to explain self-control failure [1]. A key assumption of this model is that self-control consumes a portion of one’s limited “resources” for self-control, and when these resources are depleted, the capacity for further self-regulation is reduced. Support for the strength model has been obtained in numerous experiments in which subjects were asked to accomplish consecutive self-control tasks [5]. In addition to ego-depletion, which was temporarily manipulated in the laboratory experiments noted above, researchers have found that long-term demands on self-control may impair self-control [6, 7]. For example, using a challenging counting task, Hagger et al. found that individuals with high body mass indexes (BMI), who have frequently sought to reduce their food intake, had a reduced capacity to regulate their eating under conditions of self-control resource depletion. This suggests that chronic ego depletion impairs goal-directed dieting behavior [8]. Nes et al., in an experiment in which they exposed patients diagnosed with fibromyalgia or temporomandibular disorders and pain-free matched controls to an attention control task followed by an anagram task, found that patients in the low ego-depletion condition displayed low persistence, similarly to patients and controls in the high ego-depletion condition. This result suggests that patients in chronic pain may suffer from chronic ego depletion or self-regulatory fatigue [9]. Subsequently, these researchers developed and validated a new scale, called the self-regulatory fatigue (SRF) scale, that directly measures chronic self-regulatory resource depletion [10]. While all of these studies show that chronic ego depletion impedes individuals in adhering to goals in laboratory conditions (e.g., persisting in an anagram task), the question remains of whether chronic ego depletion affects people’s goal-directed behaviors in their daily lives. Interestingly, Neal and Wood recently found that individuals with low self-control were more likely to adopt strong study habits in preparing for exams than individuals with high trait self-control (see study 5), suggesting that lower self-control can actually enhance progress toward goals furthered by strong habits [11]. A possible explanation for this finding is that habits habits are not goal-dependent and conscious behavioral regulation process [12]. In contrast to performing habits, successful execution of goal-directed behaviors depends on a wide range of cognitive, affective, and motivational processes and effortful control [13]. A recent study, employing the experience sampling method, revealed that success in resisting daily desires (a kind of goal-directed behavior) was negatively predicted by the frequency and recency of engaging in prior self-control behavior on the same day [14]. Therefore, in the current study, we predicted a negative association between chronic ego depletion and successful performance of goal directed behavior.

Although hundreds of studies support the resource model of self-control, the mechanisms by which consecutive acts of self-regulation can lead to self-regulatory collapse are widely disputed [15, 16]. For example, Muraven and Slessareva’s study revealed that motivational incentives involved in a second self-control task decrease the ego depletion effect; this finding suggests that ego depletion may derive from a motivational deficit rather than a resource deficit [17]. Furthermore, Schmeichel, Harmon-Jones, and Harmon-Jones observed that approach motivation was temporarily increased after an exercise in self-control. Failures of impulsive control may be partially explained by increased levels of reward motivation [18]. Indeed, motivation and incentives have long been known to promote the successful execution of goal directed behavior. For instance, Brehm and colleagues found that people will mobilize their energy when incentives to adhere to goals are sufficient but may fail to do so when the behavioral goal is seen as more uncertain or less important [19]. In this study, we examined the relationship between chronic ego depletion and motivation fluctuation by tracking and measuring individuals’ motivations to adhere to goals across a 3-week period. Furthermore, Inzlicht and Schmeichel proposed a process model of ego depletion, hypothesizing that previous acts of self-control cause temporary shifts in both motivation and attention that engender self-regulatory collapse [15]. They also recommended that researchers examine process variables, such as motivation to restrain and approach, attention to control, and reward cues. Therefore, using a slopes-as-outcomes multilevel analysis, we tested whether the positive relationship between motivation and behavioral performance is regulated by chronic ego depletion.

Goal directed behavior is an executive control process in which people must expend mental effort [20]. Thus, momentary effort is another import process variable of self-control. Furthermore, Muraven, Shmueli, and Burkley have found that people who expect to engage in self-control in the future have a stronger desire to conserve their limited self-control resources, especially when they have previously engaged in a self-control task [21]. That is, depleted people are more likely to withhold mental effort to conserve control resources for future demands, which may help explain why self-control suffers. In the current study, we also predicted that individuals with high chronic ego depletion reduce mental effort to engage in future goal directed behavior. In addition, previous studies have indicated that individuals who have recently suffered a loss or have fewer resources of value to defend are less likely to execute effortful control than those with greater resources [22, 23]. Hence, the potential influence of life events was controlled for in this study.

In the current study, more sophisticated methodologies than previously been used were employed to extend prior work on the nature of resource depletion effects and provide evidence with ecological validity regarding the process model of ego depletion. Overall, we tested the hypothesis that chronic ego depletion adversely affects an individual’s performance, motivation, and mental effort with respect to goal directed behavior. More importantly, we tested whether interactions among ego depletion, motivation, and mental effort can predict performance of goal directed behavior.

## Methods

### Participants and Procedure

The study was approved by the Institutional Review Board of Insitute of Psychology, Chinese Academy of Sciences (Approval number: H15003). All students signed informed consent forms approved by IRB before participating in this experiment.

To generate the target behaviors, 30 first-year graduate students were recruited to report four behaviors or life habits that they most wanted to develop. We encoded and classified their answers, and the five most frequent options were selected as the final target behaviors: keeping early hours, exercising daily, reading daily, limiting screen time, and dieting. One of these five options was selected as each participant’s behavioral goal during the formal research session.

All particpants, who were recruited from several psychology classes, first selected the behavioral goal among six options to which they most wished to adhere (see Table 1). When the number of participants reached 30 for each option, recruitment ceased. A total of 223 first-year graduate students participated in the formal research. Daily reports were returned by 208 participants, and 5 participants reported invalid response patterns, yielding a final sample of 203 participants (88 females; mean age = 23.8 years, SD = 1.20). Three invalid responders selected “keeping early hours” as their behavioral goal, and two others selected “dieting”. Of 203 valid responsers, 53 selected “keeping early hours”, 42 selected “exercising daily’, 38 selected “reading daily”, 31 selected “limiting screen time”, 28 selected “dieting”, and 11 selected “others”.

**Table 1:** Options and Operational Standards of Behavior Targets.

Options	Operational Standards ^a^
Keeping early hours	Go to bed before 11:00 PM and wake up before 7:30 AM.
Exercising daily	Spend 50 minutes jogging per day.
Reading daily	Spend 50 minutes reading per day.
Limiting screen time	Time spent on screen-based entertainment is limited to 50 minutes per day.
Dieting	Skip dinner.
Others	If your target does not fall into one of abovementioned domains, please write down a different target.

^a^ An operational standard was established for each behavior target only to keep this study reliable and repeatable. Although these operational standards are based on our previous national survey about lifestyles, they are still insufficient as international standards for healthy lifestyle.

Participants were first asked to complete a questionnaire used to reveal life events and measure chronic ego depletion. They also received a 21-daily record chart and were instructed to record their performance, perceived mental effort, and motivation to engage in goal directed behavior at the end of each day over the foollowing 3 weeks.

### Global Self-Report Measures

In this study, we used the Self-Regulatory Fatigue Scale (SRF-S) to measure participants’ levels of chronic ego depletion. We translated and amended the scale, developed by Nes, Ehlers, Whipple, and Vincent [10], to create a Chinese version [24]. The amended scale consisted of 16 items present in S1 Table. The SRF-S employed a Likert response format, with responses ranging from 1 (*not at all true*) to 5 (*very true*); higher scores reflect chronic ego depletion or a scarcity of self-regulatory resources. Cronbach’s alpha coefficient for the SRF-S was .84.

The Self-Rating Life Events Check List comprises 26 items [25]. Sample items include “I failed a recent test” and “I was rejected in love.” The effect of each negative life event occurring over the past 6 months was rated on a 5-point Likert scale from 1 (*none*) to 5 (*very severe*). Scores for all 26 items were summed to generate a total score. In this sample, Cronbach’s alpha coefficient for internal consistency was .94.

### Experience sampling measures

A three-item measure based on a scale from 0 to 10 was used to assess performance and feelings regarding goal adherence throughout the day. The items on the form read as follows: “To what extent did you fulfill your goal today?” (performance), “How much effort did you put into fulfilling your goal?” (mental effort), and “How strong is your desire to proceed with your goal?” (motivation). The participants submitted a total of 12,789 responses. We used hierarchical linear modeling (HLM) 6.0 to estimate the reliability of the experience sampling design. HLM analyses indicated an acceptable level of reliability for the performance (.88), mental effort (.96), and motivation (.96) scales.

### Data analysis methods

We used an HLM approach to evaluate covariation between chronic ego depletion and performance and feelings about goal adherence. The data were hierarchically arranged in two-level models, with 4,263 daily assessments from 203 individuals. To test the significance of the between-individual variance and the reliability of the experience sampling designs, we first estimated three null models in which no predictors were specified for either the individual- and class-level function and where the dependent variables were performance, mental effort, and motivation. After estimating the null models, we estimated a series of intercepts-as-outcome models to examine the effects of chronic ego depletion (level-2 predictor) on performance, mental effort, and motivation. To test the effects of chronic ego depletion independently, we controlled for life events and behavioral targets. Finally, to examine whether performance was predicted by daily mental effort and motivation and whether these predictive effects were regulated by chronic ego depletion, a slopes-as-outcome model was estimated. All the above-cited models included random intercepts and slopes. Level 1 variables, including mental effort and motivation, were group-mean centered, and Level 2 variables, including chronic ego depletion and life events, were grand-mean centered. Prior to these analyses, the behavioral target (a category variable) was transformed into a series of dummy variables that were added to the regression models as uncentered variables. The HLM 6.0 program was used to test the above models.

## Results

### Descriptive Statistics

Of the 203 respondents, 64 selected keeping early hours as their target, 58 selected exercising daily, 45 selected reading daily, 13 selected limiting screen time, 11 selected dieting, and 12 selected other targets. Descriptive statistics and correlations for the level-1 and level-2 variables are presented in Table 2.

**Table 2:** Correlations among the Measures of Chronic Ego Depletion, Life Events, Performance, Effort, and Motivation.

	1	2	3	4	5	Mean	*SD*
1. Chronic ego depletion	—	.19**	-.15*	.19**	-.02	32.19	8.07
2. Life events		—	.12	.12	.09	42.20	16.02
3. Behavioral performance			—	.27***	.51***	7.65	1.72
4. Mental effort				—	.22**	5.88	2.57
5. Behavioral motivation					—	8.63	1.69

Note.

* *p* < .05,

** *p* < .01,

*** *p* < .001.

### Relationships between Chronic Ego Depletion and Daily Performance, Mental Effort, and Motivation

As shown in Table 3, chronic ego depletion was inversely related to performance and positively related to mental effort. The results did not reveal a significant association between chronic ego depletion and motivation for behavior change. We also tested whether the influences of chronic ego depletion were a function of shared variance with life events or task property. With life events and targets controlled, chronic ego depletion maintained an inverse relationship with performance and a positive relationship with mental effort.

**Table 3:** Summary of Hierarchical Linear Models Predicting Daily Outcomes (Performance, Mental Effort, and Motivation).

Model predictors	Performance			Mental effort			Motivation		
	*B*	*SE*	*T*	*b*	*SE*	*t*	*b*	*SE*	*t*
Chronic ego-depletion									
Intercept	7.66	.12	63.23***	5.68	0.18	31.83***	8.60	.12	71.71***
Chronic ego-depletion	-0.03	.02	-2.16*	0.04	0.02	2.01*	0.01	.01	0.51
Chronic ego-depletion with life events and target controlled									
Intercept	7.66	.12	62.61***	5.68	0.18	31.50 ***	8.60	.12	71.51***
Chronic ego-depletion	-0.04	.02	-2.87**	0.04	0.02	1.98*	0.01	.02	0.27
Life events	0.01	.01	1.31	0.01	0.01	0.46	0.01	.01	1.17
Keeping early hours	-0.18	.59	-0.31	-0.19	0.87	-0.22	-0.19	.58	-0.32
Exercising daily	-0.19	.60	-0.33	0.03	0.88	0.03	-0.29	.59	-0.50
Dieting	-0.36	.78	-0.46	0.46	1.15	0.40	-0.12	.77	-0.16
Reading daily	-0.15	.61	-0.25	-0.35	0.90	-0.39	-0.56	.60	-0.94
Limiting online time	-0.02	.74	-0.03	-0.67	1.09	-0.62	-1.01	.72	-1.39

Note.

* *p* < .05,

** *p* < .01,

*** *p* < .001.

### Interaction Effects

We examined whether performance was predicted by daily mental effort and motivation and whether these predictive effects were regulated by chronic ego depletion. This cross-level interaction involved two within-subject predictors, daily mental effort and motivation, and one between-subject predictor, chronic ego depletion, with life events and targets controlled.

Overall, the results were not surprising: both mental effort and motivation positively predicted performance (*b* = 0.17, *SE* = .04, *t* (201) = 4.05, *p* < .001, and *b* = 0.40, *SE* = .05, *t* (201) = 8.47, *p* < .001, respectively). These results indicate that on days that the average participant exerted more mental effort and had a stronger motivation to adhere to goal directed behavior, he or she achieved better performance. The intercept was moderated by chronic ego depletion (*b* = .03, *SE* = .02, *t* (196) = -2.23, p = .014), which indicated that participants with high levels of chronic ego depletion exhibited poorer performance than those experiencing less chronic ego depletion.

**Fig 1:**
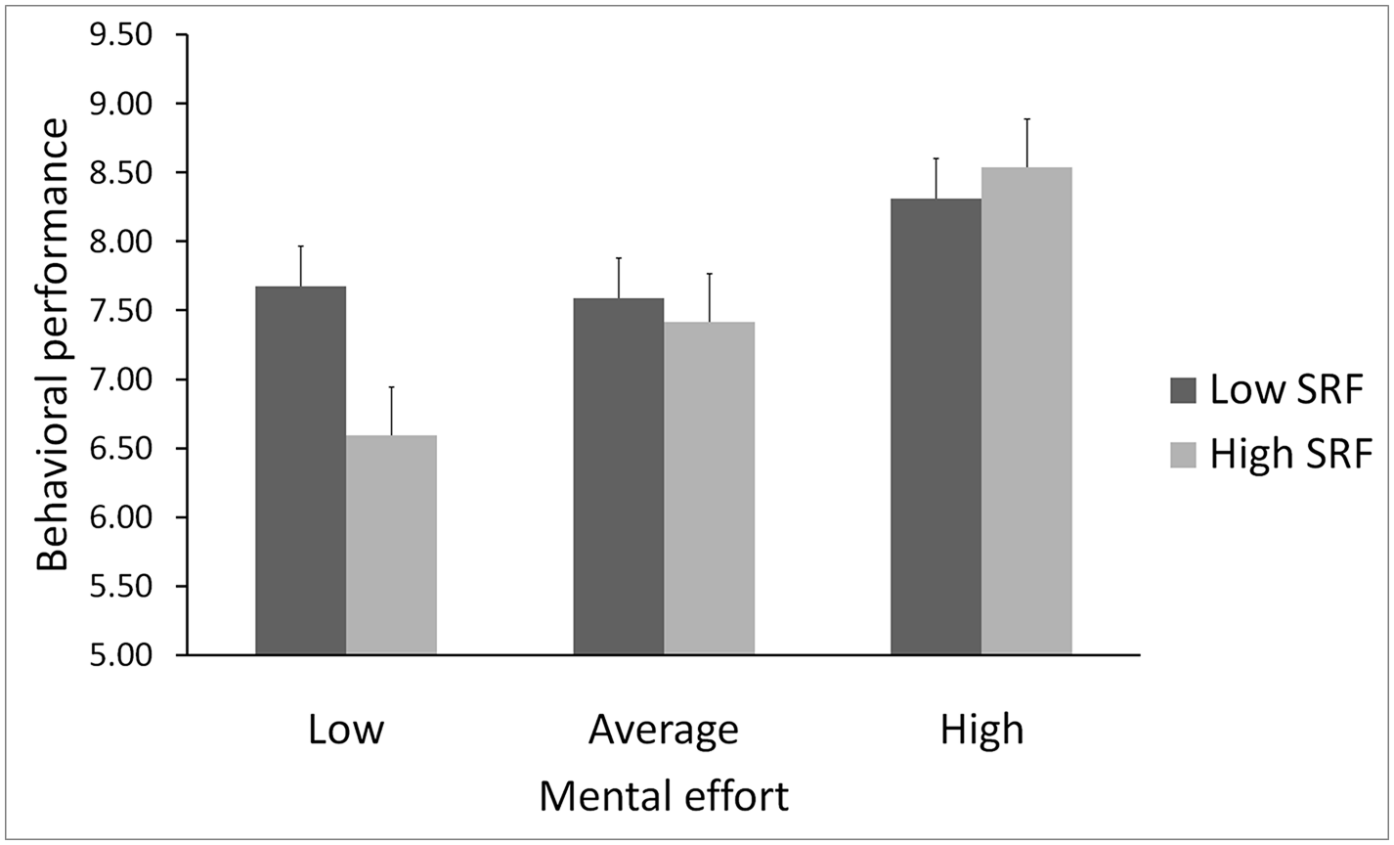
Effect of chronic ego depletion and mental effort on behavioral performance. Interaction of chronic ego depletion and daily mental effort in predicting the daily behavioral performance of individuals with at least 1 standard deviation above and below the mean on the SRF-S (1,491 daily assessments nested within 71 individuals). Low effort represents scores 1 standard deviation below the mean on the mental effort measure; average represents scores within 1 standard deviation of the mean; and high represents scores 1 standard deviation above the mean.

Testing our critical hypotheses, we observed a significant effect of chronic ego depletion on the slope for mental effort (*b* = .02, *SE* = .01, *t* (201) = 1.99, *p* = .024); however, chronic ego depletion did not moderate the slope for motivation (*b* = .01, *SE* = .01, *t* (201) = -.26, p = .791). To probe the structure of the interaction between mental effort and chronic ego depletion, the simple effects for each group were calculated and graphed following conventional procedures [26], as shown in Fig 1. Participants with high chronic ego depletion weighted mental effort heavily in their behavioral performance (slope = .41, *p* = .004), whereas the effect of daily mental effort was markedly attenuated among participants with low chronic ego depletion (slope = .24, *p* = .090). In sum, the degree to which increases in mental effort contributed to behavioral performance differed according to an individual’s level of chronic ego depletion. In particular, behavioral performance was more reliant on mental effort among participants with high chronic ego depletion.

## Discussion

Behavioral regulation or habit development is an executive control process in which people must expend mental effort [20]. In the current study, we sought to develop a more specific and nuanced understanding of self-regulatory behavior in relation to motivation, resource depletion, and mental effort.

Both acute and chronic ego depletion influence an individual’s self-control performance. Most previous studies have focused on acute ego depletion effects in consecutive self-control tasks [5], but few studies have explored chronic ego depletion inside or outside the laboratory. For example, stronger laboratorial ego depletion effects were observed among individuals engaged in long-term efforts to limit food intake [8], people with fibromyalgia and temporomandibular disorders [9], and students with high trait test anxiety [27], who were considered to be suffering from chronic ego depletion. Hofmann, Vohs, and Baumeister employed a novel operationalization of cumulative resource depletion to support the limited-resource model of self-control, finding that the frequency and recency of engaging in prior self-control negatively predicted people’s success in resisting subsequent desires on the same day [14]. Consistent with the strength model of self-control, our data also indicate that chronic ego depletion increased the likelihood of behavioral regulation failure, suggesting that individuals in an ego-depletion state have difficulty adhering to goals.

Both motivation and mental effort are process variables for self-control. Muraven and Slessareva first provided support for motivation as a key element of the ego depletion effect, observing that such depletion was overcome when participants were offered a financial incentive to perform a second self-control task [17]. However, further research by Muraven, Rosman, and Gagne failed to identify a significant difference between ego-depletion and control groups in levels of self-reported engagement and interest in a second self-control task [28]. The inconsistent results of these two studies may stem from differences in motivation types; that is, a financial incentive increases an individual’s external motivation, whereas interest is involved in internal motivation. In fact, more research attention should be focused on why people with strong internal motivation nevertheless fail to exert self-control. Therefore, we measured participants’ motivation outside the laboratory, viewing this as a form of internal motivation because participants chose the behavioral targets for themselves. Our results indicate that ego depletion did not influence people’s daily motivations to adhere to goals. Interestingly, both the correlation and HLM analysis results in this study reveal a positive association between chronic ego depletion and daily effort to adhere goals, which does not support our hypothesis. These results are consistent with motivation intensity theory [19], the core tenet of which is that effort varies proximally not with the importance of meeting a challenge but with the difficulty of meeting a challenge. When this rationale is combined with Obrist’s [29] active coping hypothesis and the idea that difficulty appraisals increase with fatigue, one can conclude that when fatigue does not change one’s belief that success is possible and worthwhile, it leads people to exert compensatory effort. In fact, some studies have found that fatigued participants experience heightened cardiovascular arousal, which is deemed an index of mental effort [30, 31]. In sum, our data did not support the view that the ego-depletion effect derives from a motivated and strategic withholding of effort.

Further evidence against the hypothesis of “shifts in motivation” [15] is that the positive association between motivation and performance was not influenced by chronic ego depletion in the current study. However, chronic ego depletion moderated the effect of mental effort on regulatory behavior; notably, performance of participants with high SRF scores relied more heavily on immediate effort than did performance of those with low SRF scores. Thus, to succeed in equally difficult tasks, people with scarce self-control resources must expend greater mental effort. We propose that in consecutive self-control tasks, the ego depletion effect arises primarily from a feeling that one should have exerted more effort than one did rather than from depleted participants withholding effort. The effect of financial incentives in Muraven and Slessareva’s [17] study in overcoming the ego depletion effect may arise because depleted participants expend more effort as a result of the additional reward for self-control at Time 2. In addition, individuals who perceived themselves as more depleted [32] or were sensitive to depletion [33] performed worse on a second self-control task, indicating a stronger ego depletion effect. Similarly, these augmented ego depletion effects may stem from a failure of participants in depleted states to exert compensatory effort.

An unfortunate limitation of this study is that we did not document participants’ perceptions of task difficulty in the daily measure. Future research is needed to determine whether perceptions of task difficulty are augmented among people with scarce self-control resources. A second limitation is that perceived self-control was not included in the daily measure; thus, we recommend that researchers explore whether the balance between perceptions of difficulty and perceptions of self-control is damaged by a recent exercise of self-control, which demands that people exert compensatory effort to recover this balance.

In conclusion, we have examined the ego depletion effect in an actual life situation and tested the process model of ego depletion. The results of the study can be summarized as follows: first, people with high levels of chronic ego depletion performed more poorly in goal adherence than people with lower levels of ego depletion; second, the daily effort devoted to goal adherence increased with chronic ego depletion, but the motivation for goal adherence was not influenced by chronic ego depletion; and third, a stronger positive association between mental effort and performance was observed among participants who scored higher on the SRF-S, while chronic ego depletion did not play a regulatory role in the effect of motivation on performance.

## Supporting Information

S1 Table Means, standard error and standard deviations for SRF-S scale items. (PDF)
