# Supplementary material for: The Influence of Chronic Ego Depletion on Goal Adherence: An Experience Sampling Study
Source: PLoS One. 2015 Nov 12;10(11):e0142220. doi: 10.1371/journal.pone.0142220 (PMC4642976; doi:10.1371/journal.pone.0142220)
Supplement: S1 Table — (PDF) [file pone.0142220.s001.pdf]

S1 Table. Means, standard error and standard deviations for SRF-S scale items.

Item	Text	Type	Mean	Standard error	Standard deviation
1	I feel full of energy	C	3.61	.060	.928
2	It's easy for me to set goals	C	3.58	.060	.928
3	I find it difficult to exercise as much as I should	C	2.82	.077	1.198
4	I have urges to hit, throw, break, or smash things	B	2.45	.084	1.301
5	I have no trouble making decisions	C	3.50	.066	1.024
6	I experience repeated unpleasant thoughts	B	2.58	.071	1.102
7	I get easily upset	E	2.43	.070	1.084
8	I try not to talk or think about things that bother me	B	2.74	.073	1.142
9	I handle stress well	E	3.59	.055	.856
10	I experience uncontrollable temper outbursts	B	2.11	.060	.929
11	I can easily keep up with my friendships and relationships	C	3.43	.064	.993
12	I cry easily	E	2.02	.071	1.102
13	I have difficulties remembering things	C	2.36	.072	1.119
14	I find it easy to stick to a healthy diet	E	3.49	.075	1.164
15	I feel moody	E	1.90	.056	.875
16	I have urges to beat, injure, or harm someone	B	1.80	.069	1.068

Abbreviations: B, behavioral; C, cognitive; E, emotional.
